# Risk stratification and management of non‐muscle‐invasive bladder cancer: A physician survey in six Asia‐Pacific territories

**DOI:** 10.1111/iju.15309

**Published:** 2023-10-06

**Authors:** Ja Hyeon Ku, Lui Shiong Lee, Tzu‐Ping Lin, Eiji Kikuchi, Hiroshi Kitamura, Chi‐Fai Ng, Junice Yi Siu Ng, Darren Ming‐Chun Poon, Ravindran Kanesvaran, Ho Kyung Seo, Carmel Spiteri, Ee Min Tan, Ben Tran, Yuh‐Shyan Tsai, Hiroyuki Nishiyama

**Affiliations:** ^1^ Seoul National University Seoul South Korea; ^2^ Seng Kang General Hospital Singapore Singapore; ^3^ Taipei Veterans General Hospital Taipei Taiwan; ^4^ St Marianna University School of Medicine Kawasaki Japan; ^5^ University of Toyama Toyama Japan; ^6^ The Chinese University of Hong Kong Hong Kong Hong Kong; ^7^ IQVIA Asia‐Pacific Singapore Singapore; ^8^ Hong Kong Sanatorium & Hospital Hong Kong Hong Kong; ^9^ National Cancer Centre Singapore Singapore Singapore; ^10^ National Cancer Center Goyang South Korea; ^11^ MSD Macquarie Park New South Wales Australia; ^12^ Peter MacCallum Cancer Centre Melbourne Victoria Australia; ^13^ National Cheng Kung University Hospital Tainan Taiwan; ^14^ University of Tsukuba Tsukuba Japan

**Keywords:** clinical practice, NMIBC, physician, survey, treatment guidelines

## Abstract

**Objectives:**

Multiple clinical practice guidelines, conflicting evidence, and physician perceptions result in variations in risk stratification among patients with non‐muscle‐invasive bladder cancer (NMIBC). This study aims to describe the extent of this variation and its impact on management approaches in the Asia‐Pacific region.

**Methods:**

We conducted a cross‐sectional survey involving 32 urologists and seven medical oncologists with ≥8 years of experience managing early‐stage bladder cancer patients across Australia, Hong Kong, Japan, South Korea, Singapore, and Taiwan. The physicians completed an anonymous questionnaire that assessed their risk stratification and respective management approaches, based on 19 NMIBC characteristics. For each NMIBC characteristic, they were required to select one risk group, and their most preferred management approach.

**Results:**

Our results demonstrated a higher consensus on risk classification versus management approaches. More than 50% of the respondents agreed on the risk classification of all NMIBC characteristics, but 42% or fewer chose the same treatment option as their preferred choice for all but two characteristics—existence of variant histology (55%) and persistent high‐grade T1 disease on repeat resection (52%). Across territories, there was the greatest variation in preferred treatment options (i.e., no treatment, intravesical chemotherapy, or Bacillus Calmette‐Guérin [BCG] treatment) for intermediate‐risk patients and the highest consensus on the treatment of very high‐risk patients, namely radical cystectomy.

**Conclusions:**

Our study revealed considerable variation in risk stratification and management of NMIBC in the region. It is critical to develop practical algorithms to facilitate the recognition of NMIBC and standardize the treatment of NMIBC patients.

**Abbreviations & Acronyms**
BCG: Bacillus Calmette‐Guérin
CIS: carcinoma in situ
CUETO: Spanish Urology Association for Oncological Treatment
EAU: European Association of Urology
EORTC: European Organization for Research and Treatment of Cancer
IRB: Institutional Review Board
IVC: intravesical chemotherapy
NMIBC: non‐muscle‐invasive bladder cancer
OS: overall survival
RC: radical cystectomy
RCT: randomized controlled trial
TURBT: Transurethral resection of bladder tumor

## INTRODUCTION

Bladder cancer is among the top 10 most prevalent cancers in the world, with over 200 000 new cases diagnosed in the Asia‐Pacific region in 2020.^1^ Non‐muscle‐invasive bladder cancer (NMIBC) is the most common form of bladder cancer and is characterized by frequent recurrence and an increased risk of progression.

Despite the availability of multiple clinical practice guidelines and consensus statements for the diagnosis and management of NMIBC patients,^2^, ^3^, ^4^, ^5^, ^6^ treatment decisions in clinical practice are frequently complicated by various patient‐related factors that influence patient prognosis and, hence, the suitability of various treatment regimens. Furthermore, discrepancies among various clinical guidelines contribute to considerable variations in the management of this disease.^7^ For instance, although a risk‐stratified approach for the management of NMIBC is recommended, the characteristics that define the various risk categories differ among guidelines.^4^, ^5^ Differences in disease management are compounded by disparities in the availability of treatment regimens across the Asia‐Pacific region.

While the NCCN‐Asia consensus statement on bladder cancer provides a brief overview of the different resources available for the management of bladder cancer in various Asian countries,^2^ there are limited data on the treatment landscape, clinical regimens, and consideration factors used by clinicians across the Asia‐Pacific region. The information on current treatment approaches for NMIBC contained in this study will help inform adherence to recommended strategies and identify management gaps. This study describes the treatment patterns employed by physicians for the management of NMIBC in the Asia‐Pacific region. A part of the results of this study was previously presented at the 19th Urological Association of Asia Congress 2022 and published as an abstract.^8^

## METHODS

### Design of questionnaire

A questionnaire was developed based on a consultative process involving 12 key opinion leaders who have extensive clinical experience in the management of early‐stage bladder cancer and are also the authors of this paper. The online questionnaire was pretested by two urologists to identify issues or ambiguities in the survey. The questionnaire contained closed‐ended questions about NMIBC management, such as the use of immediate postoperative chemotherapy. We also assessed their risk classification and management of 19 NMIBC characteristics of different risk categories. For each NMIBC characteristic, they were required to select one risk group and their most preferred management approach. A sample of the questionnaire is presented in the supplementary material. Respondents could refrain from responding if the question was not within their expertise or if they did not have sufficient experience. The questionnaire was translated into local languages and administered online.

### Eligibility criteria and respondents

Practicing urologists and medical oncologists from six territories in Asia‐Pacific (Australia, Hong Kong, Japan, South Korea, Singapore, and Taiwan) were identified from a commercial panel of clinicians who previously participated in similar surveys and were invited to participate. To be eligible, the respondents had to have at least 8 years' experience in managing patients with early‐stage bladder cancer and had spent at least 50% of time in direct patient care. As there was no a priori hypothesis, no sample size calculation was required. The respondents were only made aware of the study sponsor at the end of data collection.

### Data analysis and interpretation

Descriptive analysis, namely count and frequencies, was performed. Questions in which proportions were indicated for corresponding options were analyzed by rank to determine the most frequently used options. Due to the opt‐out options, the total number of responses varied depending on the question. No statistical tests were conducted beyond descriptive statistics, as no hypothesis was tested.

## RESULTS

### Characteristics of respondents

Of 55 clinicians who were approached and responded, 40 participated in the study. One respondent was excluded due to involvement as an advisor of the study and 39 respondents were included in the analysis. A majority of the participants were urologists (82%), as shown in Table 1.

**TABLE 1:** Characteristics of respondents (*N* = 39).

Characteristics	*n* (%)
Country/region	
Australia	8 (21)
Hong Kong	3 (8)
Japan	9 (23)
Korea	8 (21)
Taiwan	6 (15)
Singapore	5 (13)
Specialty	
Urologist	32 (82)
Medical oncologist	7 (18)
Practice	
Public only	17 (44)
Private only	20 (51)
Both public and private	2 (5)

### Use of immediate postoperative intravesical chemotherapy regimen

Approximately 64% of respondents (21/33) indicated that they would consider immediate intravesical chemotherapy (IVC) after transurethral resection of the bladder tumor (TURBT), i.e. single instillation of IVC within 24 h of TURBT, in majority of newly diagnosed NMIBC patients. 33% of respondents (11/33) would consider IVC use in a smaller proportion of their patients, whereas one participant indicated equal proportions of patients would or would not receive immediate post‐TURBT IVC. Three respondents from Korea and one from Australia indicated that immediate IVC was not used in any of their patients. In those who used IVC, the most preferred chemotherapy agent in this setting was mitomycin (59%), followed by epirubicin (21%) (Table 2).

**TABLE 2:** Preferred chemotherapy agents among respondents who would generally use immediate intravesical chemotherapy (IVC) post‐TURBT in newly diagnosed NMIBC patients.

Preferred chemotherapy agents among respondents who would generally use single instillation of IVC post‐TURBT (*N* = 29)^a^	*n* (%)
Mitomycin C	17 (59)
Epirubicin	6 (21)
Gemcitabine	4 (14)
Pirarubicin	2 (7)

Abbreviations: IVC, intravesical chemotherapy; TURBT, transurethral resection of the bladder tumor.

^a^ 6 physicians did not respond to this question and 4 respondents indicated that they do not use immediate postoperative IVC in any of their patients.

### Risk stratification and management

Among 36 respondents, 56% used the European Organization for Research and Treatment of Cancer (EORTC) risk table; 25% assessed patients' risk of progression subjectively, 3% used the Spanish Urology Association for Oncological Treatment (CUETO) risk table and 14% used other tools (Table S1). Three respondents did not perform risk classification.

**FIGURE 1:**
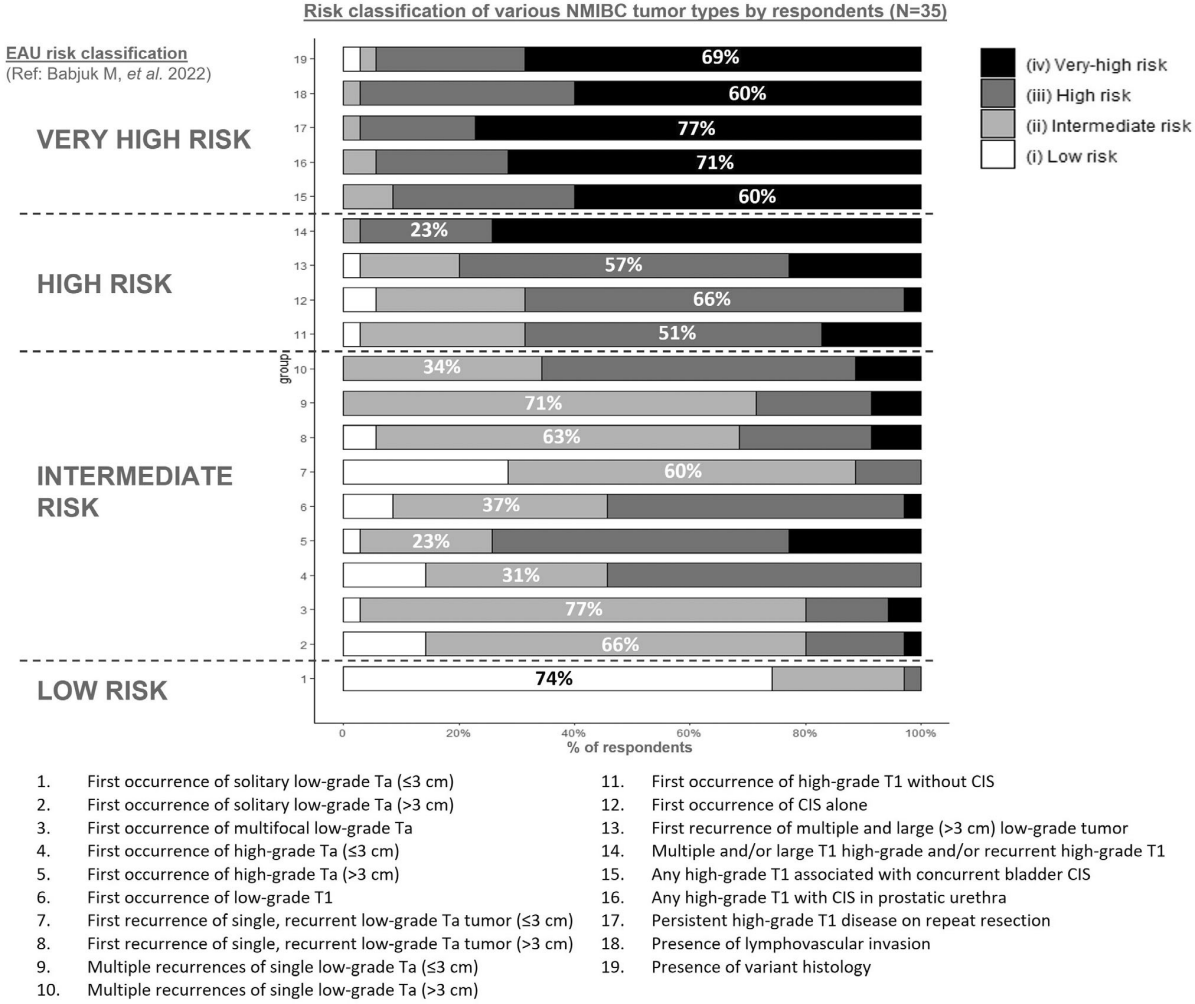
Most commonly selected risk categories for indicated NMIBC characteristics, in comparison with the EAU risk classification, among urologists and medical oncologists (*N* = 35) in Asia Pacific. Four physicians did not respond to this question.

**FIGURE 2:**
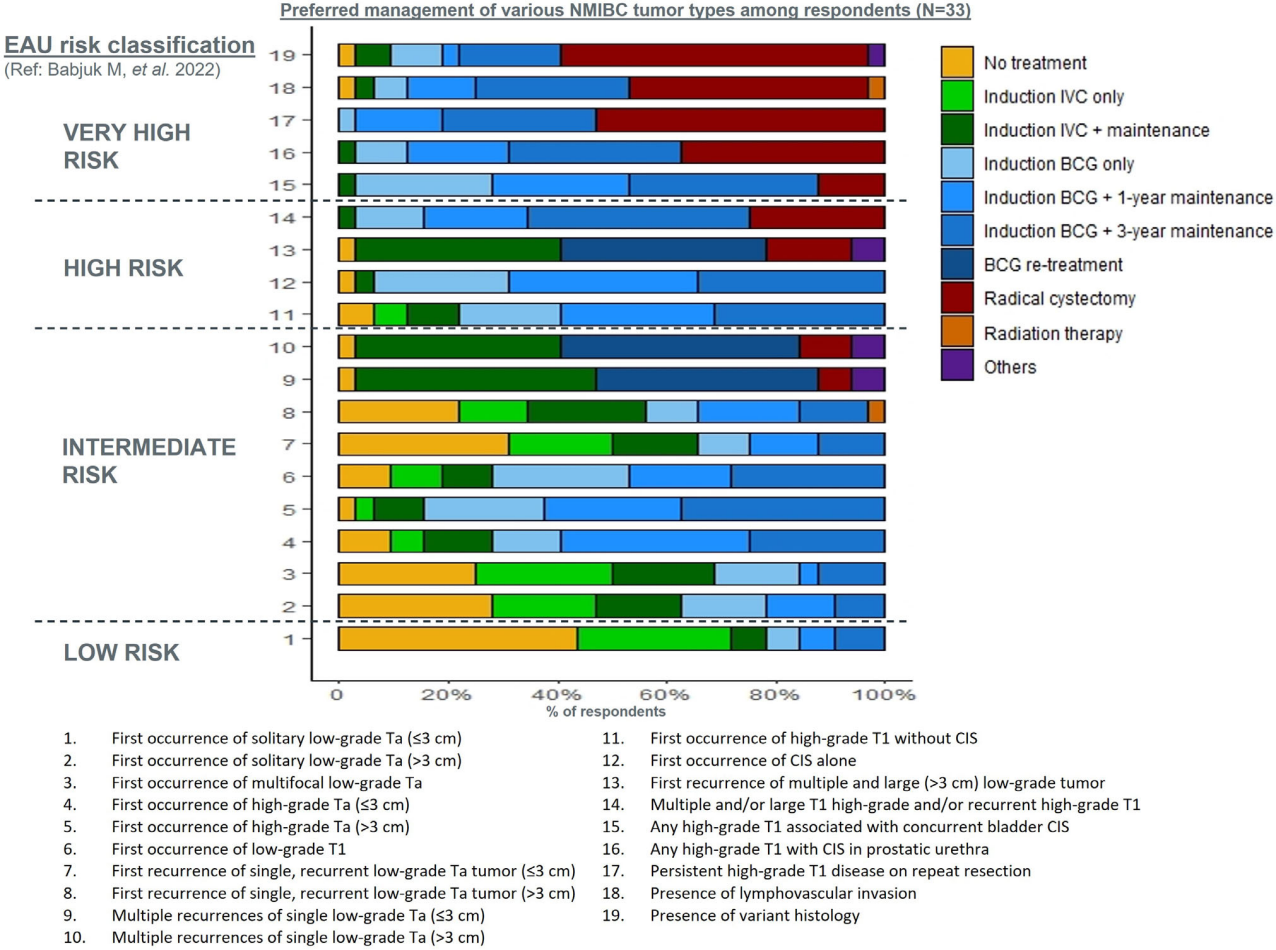
Most commonly preferred management options for NMIBC characteristics among urologists and medical oncologists (*N* = 33) in Asia‐Pacific. Other options included second TURBT, pirarubicin and management dependent on variant histology. Six physicians did not respond to this survey question. BCG, Bacillus Calmette‐Guerin; IVC, intravesical chemotherapy.

Figure 1 shows respondents' risk classification of 19 NMIBC characteristics, while Figure 2 shows corresponding preferred treatment options. There was a higher consensus on risk classification of NMIBC characteristics versus management approaches for NMIBC. More than 50% of the respondents selected the same risk category for all characteristics. In contrast, only two characteristics—persistently high‐grade T1 disease on repeat resection and existence of variant histology (Figure 2, #17 and #19)—were treated using the same approach (radical cystectomy [RC]) by more than 50% of the respondents.

In general, except for multiple and recurrent large (>3 cm) tumors, low‐grade Ta non‐muscle invasive tumors were considered intermediate‐risk (Figure 1). Multiple recurrences of large (>3 cm) low‐grade Ta, low‐grade T1, high‐grade Ta and T1 (without carcinoma in situ [CIS]), and CIS alone were considered high‐risk prognostic factors, and CIS with high‐grade T1, existence of lymphovascular invasion, and variant histology were considered very high‐risk factors. The respondents were more likely to suggest no treatment for low‐risk and intermediate‐risk patients, intravesical Bacillus Calmette‐Guérin (BCG) for high‐risk patients, and RC for very high‐risk patients (Figure 2).

For the first recurrence of single, recurrent low‐grade Ta tumors (both ≤3 cm and >3 cm), main management approaches included observation only, induction BCG with one‐year maintenance, and induction IVC with maintenance (Figure 2, #7 and #8).

Administering IVC with maintenance and rechallenging with BCG were preferred management approaches for multiple recurrences of single low‐grade Ta (both ≤3 cm and >3 cm), after receiving adequate BCG (Figure 2, #9 and #10). For very‐high risk characteristics, induction BCG with maintenance and RC were preferred.

### Reasons for using adjuvant IVC

**FIGURE 3:**
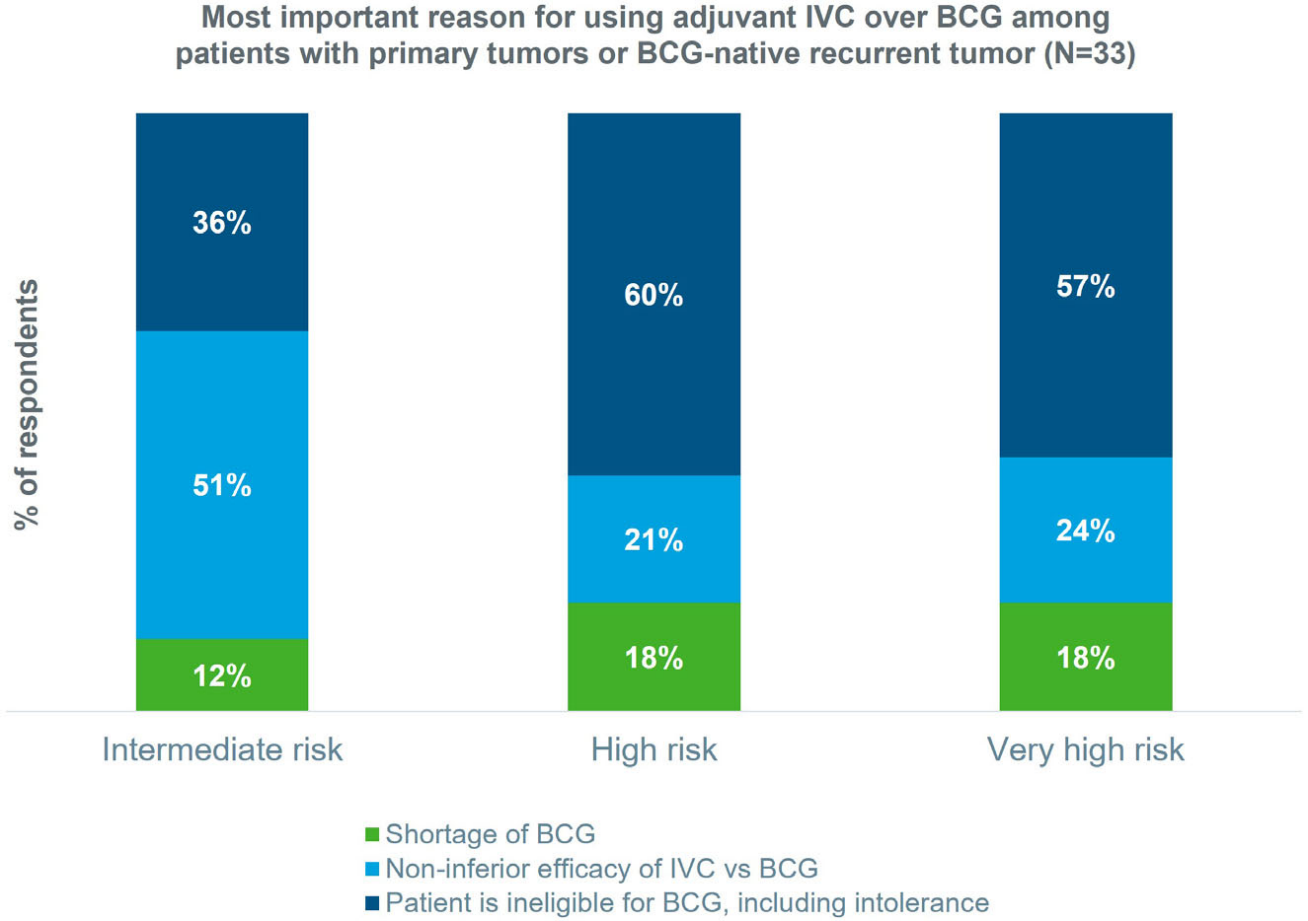
Most important reason for using adjuvant IVC over BCG among patients with primary tumors or BCG‐naïve recurrent tumor among urologists and medical oncologists (*N* = 33) in Asia‐Pacific. Six physicians did not respond to this survey question. Adjuvant IVC is immediate post‐TURBT IVC and/or later repeat instillations. Risk group of patients as defined by respondents in their usual practice. BCG, Bacillus Calmette‐Guerin; IVC, intravesical chemotherapy.

For intermediate‐risk patients, 51% of the respondents used adjuvant IVC, that is, immediate post‐TURBT IVC and/or later repeat instillations, because of the noninferior efficacy of IVC versus BCG (Figure 3). In contrast, patient ineligibility for BCG was the key reason for using adjuvant IVC for high‐risk (60%) and very high‐risk (57%) patients.

### BCG instillations and schedule

The most commonly used definition of adequate BCG is six out of six instillations (42%) for the initial induction cycle, two out of three instillations (48%) for maintenance, and six out of six instillations (33%) for the second induction cycle (Table S2).

Regarding the use of BCG maintenance, the first‐year maintenance therapy was prioritized over the second‐ and third‐year maintenance therapy during BCG shortage (Figure S1). This is demonstrated by the smaller difference in the median number of weekly instillations during BCG shortage relative to no BCG shortage across all risk groups in the first year, compared with that in the second and third years. The number of weekly instillations at three, six, and 12 months was reduced for intermediate‐risk patients during BCG shortage but remained the same for high‐risk and very high‐risk patients. This suggests that during BCG shortage, physicians were more likely to ration the use of BCG for intermediate‐risk patients and maintain the same number of weekly instillations at three, six, and 12 months at full dose for high‐ and very high‐risk patients (Figure S2).

## DISCUSSION

In this study, considerable practice variations were observed among clinicians in the Asia‐Pacific region regarding management of NMIBC tumors. There was greater concordance in risk classification versus management of NMIBC. This was possibly due to factors beyond risk classification, such as patient tolerance to BCG, BCG supply, and physician confidence in the completeness of TURBT. In addition, a wider range of treatment approaches was proposed for intermediate‐risk patients when compared to high‐risk and very high‐risk patients, possibly reflecting how intermediate‐risk patients represent a heterogeneous population. This study also shows that clinicians prioritize higher‐risk patients for use of BCG in times of BCG shortages, an observation that is in line with consensus recommendations.^9^

Variability in risk classification was greatest in our study for high‐grade Ta, low‐grade T1, recurrences of low‐grade Ta, and recurrences of multiple, large low‐grade tumors. Similarly, a survey conducted in Europe among 498 physicians also demonstrated higher variability in the risk stratification of T1, multiple and recurrent TaG1‐2, and recurrent tumors.^10^ Variations in risk classification may result from the use of different risk classification tools or guidelines. In practice, the management of T1 tumors is commonly guided by pathological findings from a second TURBT.^11^ When considering treatment of NMIBC, it is necessary to consider that repeat TURBT may affect the accurate diagnosis and risk of recurrence. Prevalence and consensus regarding new TURBT techniques such as second TURBT and photodynamic diagnosis‐assisted TURBT should be examined in the future. The timing of recurrence and prior treatments used were not accounted for in our study, which might have affected treatment decisions. The respondents were also polled on their preferred treatment options, which might not have translated into their clinical practice, such as the discontinuation of BCG therapy due to complications despite the preference for maintenance treatment. Clinicians' compliance with guideline recommendations in this study shows that there is variation among clinicians in adopting different recommendations for the same risk stratification and disease characteristics.

A review of current literature and clinical practice guidelines on intermediate‐risk NMIBC found that this is a heterogeneous group, typically comprising patients excluded from being classified as low‐risk or high‐risk NMIBC.^12^ Furthermore, there is a lack of studies investigating therapies and outcomes in intermediate‐risk patients. This could explain the diverse treatment approaches selected by the respondents in our study. Although the International Bladder Cancer Group has introduced a simpler algorithm to sub‐stratify this group of patients into “similar to low‐risk,” “intermediate‐risk,” and “similar to high‐risk,” based on the number of risk factors, such an approach adds to the complexity of risk classification.

Although guidelines recommend BCG should be given in a maintenance schedule for high‐risk patients,^5^ our study revealed that approximately a third of respondents selected induction BCG only, induction BCG with one‐year maintenance and induction BCG with three‐year maintenance for these patients. This could be due to conflicting evidence on the effectiveness of BCG maintenance therapy of varying duration. A network meta‐analysis of approximately 2000 patients found that longer BCG maintenance therapy of up to 3 years was not superior to shorter one‐year maintenance therapy in reducing tumor recurrence and the progression rate of NMIBC; however, maintenance therapy overall was better than induction‐only BCG therapy while not increasing side effects.^13^ On the other hand, in a randomized controlled trial (RCT) conducted among 1355 patients, EORTC showed that when BCG was given at full dose, three‐year maintenance reduces the recurrence rate when compared to one‐year maintenance in high‐risk, but not intermediate‐risk patients.^14^ There was no significant difference in the progression rate or overall survival (OS). A retrospective non‐randomized comparative study also found that non‐maintenance eight‐dose induction BCG was inferior to six‐dose induction BCG plus 36‐month maintenance BCG in high‐risk and very‐high risk patients in recurrence risk reduction. Nevertheless, the former might be an alternative remedy in the era of BCG shortages.^15^

Our findings showed that less than five clinicians preferred providing IVC to high‐risk and very high‐risk patients with primary tumors. This corroborates the findings of a survey conducted among European physicians, in which IVC was given to 9%–30% of high‐risk patients.^10^ Another study involving 774 high‐risk patients in North America and Europe also found that only 12.5% received IVC.^16^ The key reasons for preferring IVC over BCG for primary tumors were in line with our understanding (ie, BCG shortage and ineligibility to BCG). Surprisingly, our study also revealed clinicians' perception of noninferior efficacy of IVC in high‐risk and very high‐risk NMIBC and thus its role in the treatment of these patients. A recent systematic review of 12 RCTs comparing BCG versus mitomycin C in patients with intermediate‐risk and high‐risk NMIBC tumors found that BCG might have improved time‐to‐recurrence but might not have impacted time‐to‐mortality from any cause, or time‐to‐progression among 2932 participants.^17^ However, the certainty of this evidence remains low due to selection bias and the lack of blinding in these studies.

In our study, more respondents preferred RC to induction BCG with maintenance for very high‐risk patients. While this is in agreement with clinical practice guidelines,^2^, ^3^, ^4^, ^5^, ^6^ it is unclear if this translates into a higher proportion of patients receiving RC in clinical practice. The BRAVO feasibility study was the first head‐to‐head trial investigating the efficacy of RC versus bladder‐sparing treatment options.^18^ At study follow‐up, all patients in the RC arm were free of disease, while two patients in the BCG arm had metastatic disease. However, the study was underpowered to draw any definitive conclusions. A meta‐analysis of cohort studies comprising 1735 patients demonstrated that compared with RC, the bladder preservation group had improved 10‐year OS (odds ratio 0.62 [95% confidence interval 0.43–0.88, *p* = 0.007, *I* ^2^ = 0%]).^19^ The authors concluded that bladder preservation is a superior treatment modality compared to RC, especially for older patients and for those with T1G3 or lower‐grade tumors. Conversely, RC could be a better option for younger patients. In clinical practice, other than effectiveness, the clinician also weighs other factors, such as patients' willingness to accept RC, their quality of life after surgery (especially for elderly patients), side effects, and cost. Patient factors including age, gender should also be considered in the management of patients with NMIBC and based on the risk–benefit assessment, an individualized risk‐adaptive approach can be developed.

Our study is dependent on self‐report by clinicians and may be limited by clinicians reporting different practices than those used, providing a better representation of their attitudes than practiced. The small sample size is not statistically representative of all clinicians in Asia‐Pacific, but the coverage of the relevant medical specialties and work settings suggests that this study is still informative regarding management approaches to NMIBC tumors in this region. Another limitation is the low participation rate, as the physicians selected may not be fully representative of their territories. However, to the best of our knowledge, this study is one of the few providing insights into the management of NMIBC patients in the region. This study was conducted in six territories in Asia‐Pacific, taking into the prevalence of BCG and other treatment agents. The results of our study could help inform a larger‐scale region‐wide survey in the future. One of the main strengths of this study is the detailed information collected from physicians' perspective, including perceived risk assessment and suggested management approaches. This allowed the identification of actionable items, such as producing more robust evidence that might improve the management of NMIBC in real‐world settings.

Our analysis of the clinician perspective in the management of NMIBC showed considerable variations in practice across the Asia‐Pacific region. Since therapeutic decisions are largely based on risk stratification, it is critical to develop practical algorithms to facilitate the recognition of NMIBC and to standardize the treatment of NMIBC patients at risk of progression.

## AUTHOR CONTRIBUTIONS

**Ja Hyeon Ku:** Conceptualization; Writing—review & editing; Methodology; Validation. **Lui Shiong Lee:** Conceptualization; Writing ‐ review & editing; Methodology. **Tzu‐Ping Lin:** Conceptualization; Writing—review & editing; Methodology. **Eiji Kikuchi:** Conceptualization; Methodology; Writing—review & editing. **Hiroshi Kitamura:** Conceptualization; Methodology; Writing—review & editing. **Chi‐Fai Ng:** Conceptualization; Methodology; Writing—review & editing. **Junice Yi Siu Ng:** Formal analysis; Writing—original draft; Project administration; Writing—review & editing. **Darren Ming‐Chun Poon:** Conceptualization; Methodology; Writing—review & editing. **Ravindran Kanesvaran:** Conceptualization; Methodology; Writing—review & editing. **Ho Kyung Seo:** Conceptualization; Methodology; Writing—review & editing. **Carmel Spiteri:** Conceptualization; Methodology; Writing—review & editing. **Ee Min Tan:** Formal analysis; Writing—original draft; Writing—review & editing; Project administration. **Ben Tran:** Conceptualization; Methodology; Writing—review & editing. **Yuh‐Shyan Tsai:** Conceptualization; Methodology; Writing—review & editing. **Hiroyuki Nishiyama:** Conceptualization; Methodology; Supervision; Writing—review & editing.

## CONFLICT OF INTEREST STATEMENT

LLS received research funding from Janssen, honoraria from AstraZeneca, MSD, Bayer, Astellas and Janssen; played an advisory role for AstraZeneca, MSD, Bayer, Astellas and Janssen. LTP received honoraria from Astellas, MSD, Ferring pharmaceuticals, Janssen, Bayer and AstraZeneca; received Docetaxel/Cabazitaxel compounds from Sanofi. KE received consulting fees and honoraria from MSD. KH received consulting fees from Kissei and Takeda; received honoraria from Astellas, AstraZeneca, MSD, Sanofi, Takeda, Bayer, Bristol Myers Squibb, Merck Biopharma and Janssen; played an advisory role for Janssen, Astellas, MSD and Pfizer. NCF received consulting fees from Cornerstone Medical and Agilis; received honoraria from Boston Scientific, Janssen, Olympus, Bayer and Ipsen; played an advisory role for Newlife Medical Limited, Jiangsu Hengrui Medicine and Nonagen. He also received equipment materials/drugs from Olympus and Janssen. KR received consulting fees from MSD, AstraZeneca, Bristol‐Myers Squibb, Eisai, Astellas, Johnson & Johnson and Pfizer; received honoraria from MSD, AstraZeneca, Bristol‐Myers Squibb, Astellas, Pfizer and Johnson & Johnson. TB received research fundings Bristol‐Myers Squibb, MSD, Ipsen, Astellas Pharma, Janssen‐Cilag, Amgen, Pfizer, Genentech, AstraZeneca and Bayer. He received honoraria from Astellas Pharma, MSD, Janssen‐Cilag, Sanofi, Tolmar, Amgen, Bristol‐Myers Squibb, Pfizer, Janssen and Bayer; received travel grants from Amgen and Astellas Pharma; played an advisory role for Amgen, Astellas Pharma, Bayer, Sanofi, Tolmar, Janssen‐Cilag, Bristol‐Meyers Squibb, Ipsen, MSD Oncology, IQVIA, Novartis, Pfizer/EMD Serono, AstraZeneca and Roche Molecular Diagnostic. NH received research grants from Chugai Pharmaceutical and Ono Pharmaceutical; received honoraria from MSD, Astellas, Bristol Myers Squibb, Pfizer/Merck Biopharma and Nihonkayaku; played an advisory role for AstraZeneca, MSD, Astellas, Chugai Pharmaceutical, Ono Pharmaceutical and Janssen. NJYS and TEM were full‐time employees of IQVIA, that was commissioned to carry out this study. SC was a full‐time employee of MSD. KJH, PDMC, SHK, TYS received honoraria from MSD and have no other conflicts of interest to declare. This work was supported by funding from MSD International GmBH (Singapore). The funding source had no role in the analysis of this study.

## APPROVAL OF THE RESEARCH PROTOCOL BY AN INSTITUTIONAL REVIEWER BOARD

This protocol for this research project conforms to the provisions of the Declaration of Helsinki and has been approved by the following research ethics committee: Melbourne Health Human Research Ethics Committee (HREC/75511/MH‐2021), Hong Kong Society of Uro‐Oncology Medical Council, St Marianna's University Ethics Board, Seoul National University Hospital Institutional Review Board (IRB) (H‐2104‐053‐1210), SingHealth Centralized IRB (2021/2124) and Taipei Veterans General Hospital IRB (2021‐06‐002BC).

## INFORMED CONSENT

Informed consent was obtained from all the respondents.

## REGISTRY AND THE REGISTRATION NO. OF THE STUDY/TRIAL

N/A.

## ANIMAL STUDIES

N/A.

## Supporting information


Table S1

Table S2

Figure S1

Figure S2

Data S1
