# Supplementary material for: Risk stratification and management of non‐muscle‐invasive bladder cancer: A physician survey in six Asia‐Pacific territories
Source: Int J Urol. 2023 Oct 6;31(1):64–71. doi: 10.1111/iju.15309 (PMC11524120; doi:10.1111/iju.15309)
Supplement: Supplementary file 1 — Table S1 [file IJU-31-64-s003.docx]

**Supplemental Table 1.** Assessment of risk among urologists and medical oncologists (N=36) in Asia-Pacific.

| Risk tables or calculators (N = 36)^a,b^ | n (%) |
| --- | --- |
| EORTC risk table | 20 (56) |
| Perform risk stratification, but do not use risk table/calculator | 9 (25) |
| CUETO risk table | 1 (3) |
| Others^c^ | 5 (14) |
| Do not perform risk stratification | 3 (8) |

^a^3 physicians did not respond to this question

^b^Respondents could select more than one option*.*

^c^Others include AUA Risk Stratification (n = 2), EAU Risk Stratification (n = 2), and guideline by Japan Urology Association (n = 2)

CUETO: Spanish Urology Association for Oncological Treatment; EORTC: European Organization for Research and Treatment of Cancer
