# Supplementary material for: Risk stratification and management of non‐muscle‐invasive bladder cancer: A physician survey in six Asia‐Pacific territories
Source: Int J Urol. 2023 Oct 6;31(1):64–71. doi: 10.1111/iju.15309 (PMC11524120; doi:10.1111/iju.15309)
Supplement: Supplementary file 2 — Table S2 [file IJU-31-64-s002.docx]

**Supplemental Table 2.** Minimum number of instillations considered as adequate BCG treatment by urologists and medical oncologists in Asia-Pacific

| BCG instillations for induction/ maintenance (N = 33)^a^ | n (%) |
| --- | --- |
| Initial induction cycle |  |
| 6 out of 6 | 14 (42) |
| 4 out of 6 | 8 (24) |
| 5 out of 6 | 6 (18) |
| Others^b^ | 5 (15) |
|  |  |
| Maintenance |  |
| 2 out of 3 | 16 (48) |
| 0 out of 0 | 2 (6) |
| 1 out of 1 | 2 (6) |
| 1 out of 3 | 2 (6) |
| 3 out of 6 | 2 (6) |
| Others^c^ | 9 (27) |
|  |  |
| Second induction cycle |  |
| 6 out of 6 | 11 (33) |
| 4 out of 6 | 4 (12) |
| 2 out of 6 | 3 (9) |
| 3 out of 6 | 3 (9) |
| 5 out of 6 | 3 (9) |
| 6 out of 8 | 3 (9) |
| Others^d^ | 6 (18) |

| BCG: Bacillus Calmette-Guerin  ^a^6 physicians did not respond to this question  ^b^Others comprise 2 out of 6 (n = 1), 3 out of 6 (n = 1), 4 out of 8 (n = 1) and 6 out of 8 (n = 2). |
| --- |
| ^c^Other comprise 2 out of 6 (n = 1), 3 out of 4 (n = 1), 3 out of 5 (n = 1), 3 out of 7 (n = 1), 4 out of 6 (n = 1), 6 out of 6 (n = 1), 6 out of 8 (n = 1), 6 out of 9 (n = 1) and 9 out of 9 (n = 1). |
| ^d^Others comprise 0 out of 0 (n = 1), 1 out of 3 (n = 2), 2 out of 3 (n = 1) and 3 out of 3 (n = 2). |
