# Supplementary figures and images for: Risk stratification and management of non‐muscle‐invasive bladder cancer: A physician survey in six Asia‐Pacific territories
Source: Int J Urol. 2023 Oct 6;31(1):64–71. doi: 10.1111/iju.15309 (PMC11524120; doi:10.1111/iju.15309)

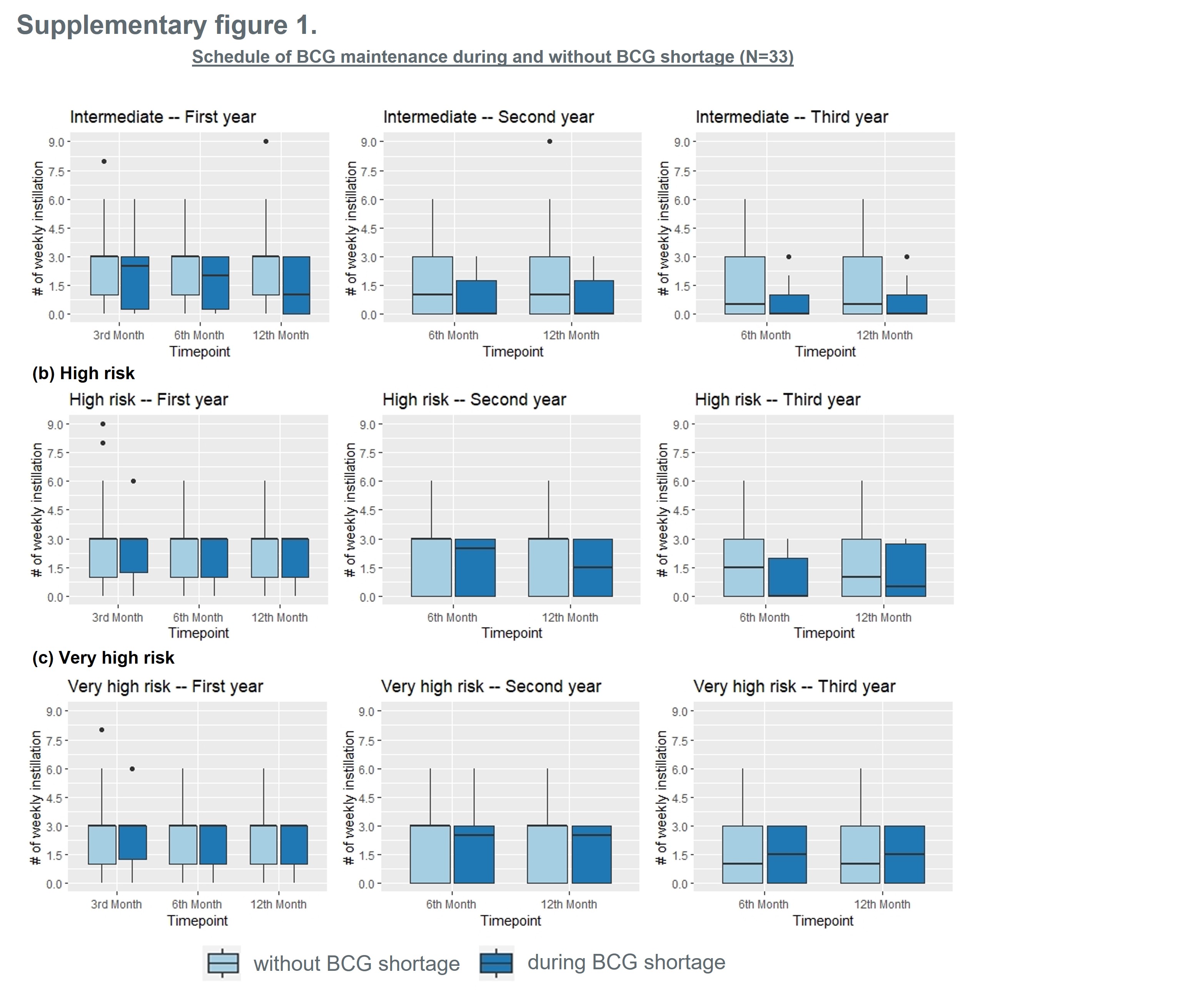

Supplement: Supplementary file 3 — Figure S1 [file IJU-31-64-s004.jpg]

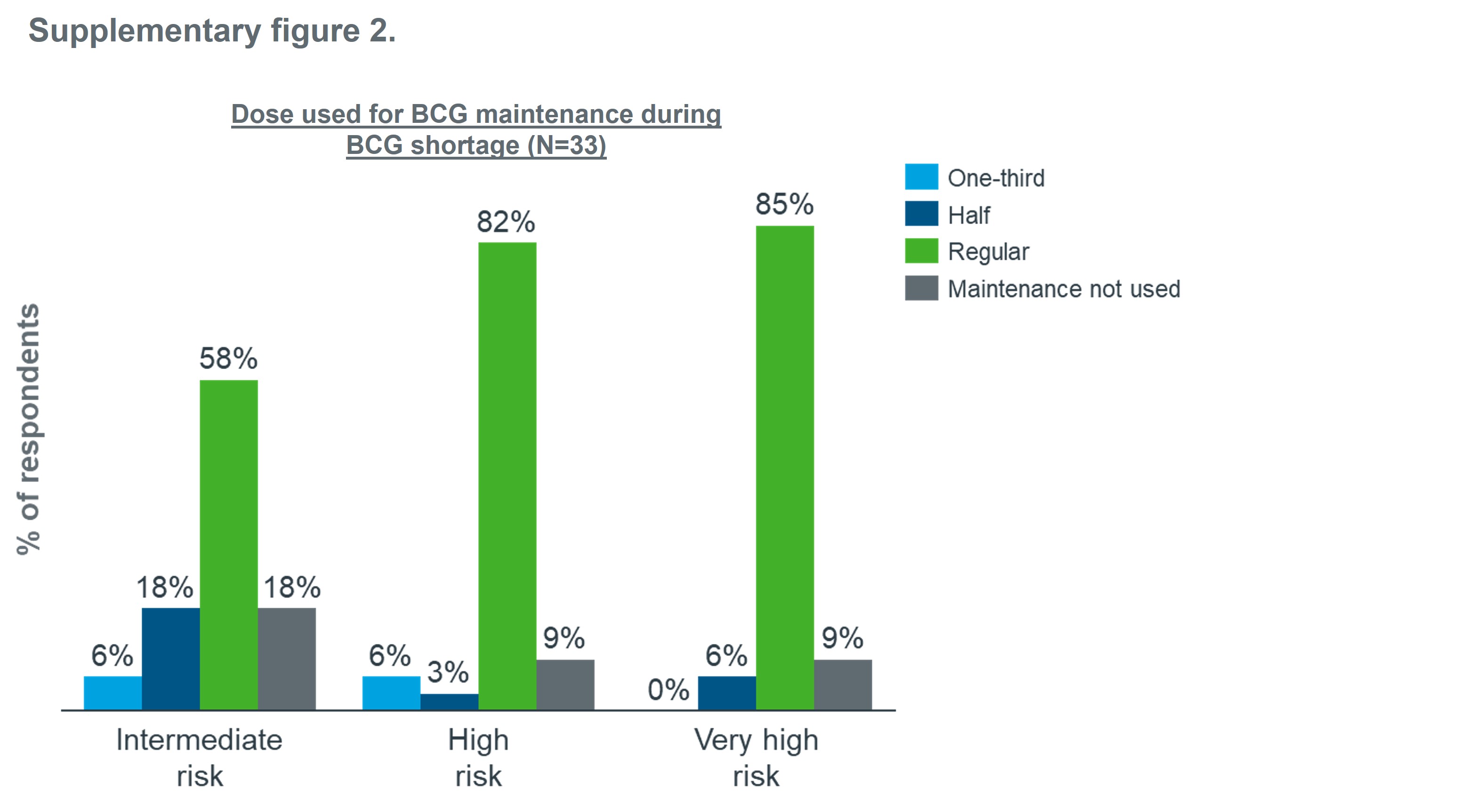

Supplement: Supplementary file 4 — Figure S2 [file IJU-31-64-s005.jpg]
