# Supplementary material for: Risk stratification and management of non‐muscle‐invasive bladder cancer: A physician survey in six Asia‐Pacific territories
Source: Int J Urol. 2023 Oct 6;31(1):64–71. doi: 10.1111/iju.15309 (PMC11524120; doi:10.1111/iju.15309)
Supplement: Supplementary file 5 — Data S1 [file IJU-31-64-s001.docx]

**Supplemental Material**

Sample questionnaire

1. Please indicate the proportion of NMIBC patients (per year) with newly-diagnosed tumor(s) that receive the following **immediate postoperative intravesical chemotherapy** regimen (single instillation post-TURB) in your practice.

**For medical oncologists**

 Opt-out from answering this question as I do not treat many of these patients in my practice

 Opt-out from answering this question as not within my clinical expertise/ not qualified

| **Option** | % of patients with **newly-diagnosed NMIBC** tumor(s) that receive this treatment |
| --- | --- |
| - 1. Single instillation of chemotherapy within 24h of TURB |  |
| 1. Immediate postoperative chemotherapy not used |  |
| 1. Others, please specify |  |
| Total | 100% |

1. Please choose up to three preferred chemotherapy options for **immediate postoperative intravesical chemotherapy** (single instillation post-TURB) in NMIBC patients. Please indicate “1” for the preferred first choice, “2” for the second choice (if applicable) and “3” for the third choice (if applicable). You do not have to indicate all three preferences if fewer than three preferred options are used in your practice.

**For medical oncologist**

 Opt-out from answering this question as I do not treat many of these patients in my practice

 Opt-out from answering this question as not within my clinical expertise/ not qualified

| **Option** | Preferred immediate postoperative intravesical chemotherapy options for patients with **newly-diagnosed** NMIBC tumor(s) |
| --- | --- |
| 1. Gemcitabine |  |
| 1. Mitomycin C |  |
| 1. Epirubicin |  |
| 1. Doxorubicin |  |
| 1. Others, please specify |  |

1. Which of the following risk tables/ calculators do you use to predict recurrence and progression in your practice? Please select all options that are relevant.

**For medical oncologist**

 Opt-out from answering this question as I do not treat many of these patients in my practice

 Opt-out from answering this question as not within my clinical expertise/ not qualified

| I risk-stratify but do not use risk table/calculator | 1 |
| --- | --- |
| EORTC risk table | 2 |
| CUETO risk table | 3 |
| I do not perform risk classification | 4 |
| Others, please specify | 5 |

1. Please **rank** the following reasons for using **adjuvant intravesical chemotherapy** in your respective NMIBC patients in each column, with **“1”** being the **most important** reason and **“4”** being the **least important** reason. An example is shown below.

**For medical oncologist**

🖵 Opt-out from answering this question as I do not treat many of these patients in my practice

🖵 Opt-out from answering this question as not within my clinical expertise/ not qualified

| **Reasons** | ***E.G. Intermediate-risk*** | **(i) Intermediate-risk** | **(ii) High-risk** | **(iii) Very-high-risk** |
| --- | --- | --- | --- | --- |
| 1. Shortage of BCG | *4* |  |  |  |
| 1. Patient is ineligible for BCG | *3* |  |  |  |
| 1. Non-inferior efficacy of intravesical chemotherapy compared with BCG in these patients | *1* |  |  |  |
| 1. Better tolerability of intravesical chemotherapy over BCG in these patients (despite eligibility for BCG) | *2* |  |  |  |

1. Please indicate below how you would classify the following characteristics of patients with NMIBC in your practice, by selecting the circle in the corresponding column. Please select only one circle per row. An example is shown in the first row.

**For medical oncologist**

🖵 Opt-out from answering this question as I do not treat many of these patients in my practice

🖵 Opt-out from answering this question as not within my clinical expertise/ not qualified

| **Characteristics** | **(i) Low risk** | **(ii) Intermediate risk** | **(iii) High risk** | **(iv) Very-high risk** |
| --- | --- | --- | --- | --- |
| *E.g. Solitary low-grade Ta >3 cm* | *🗸* |  |  |  |
| 1. First occurrence of solitary low-grade Ta >3 cm |  |  |  |  |
| 1. First occurrence of solitary low-grade Ta ≤3 cm |  |  |  |  |
| 1. First occurrence of low-grade Ta, multifocal |  |  |  |  |
| 1. First occurrence of high-grade Ta, ≤3 cm |  |  |  |  |
| 1. First occurrence of high-grade Ta, >3 cm |  |  |  |  |
| 1. First occurrence of low-grade T1 |  |  |  |  |
| 1. First occurrence of high-grade T1 without CIS |  |  |  |  |
| 1. First occurrence of CIS alone |  |  |  |  |
| 1. First recurrence of single, recurrent low-grade Ta tumor (≤3 cm) |  |  |  |  |
| 1. First recurrence of single, recurrent low-grade Ta tumor (>3 cm) |  |  |  |  |
| 1. Single low-grade Ta (≤3 cm), multiple recurrences |  |  |  |  |
| 1. Single low-grade Ta (>3 cm), multiple recurrences |  |  |  |  |
| 1. First recurrence of multiple and large (>3 cm) low-grade tumor (all features present) |  |  |  |  |
| 1. Any high-grade T1 associated with concurrent bladder CIS |  |  |  |  |
| 1. Any high-grade T1 with CIS in the prostatic urethra |  |  |  |  |
| 1. Multiple and/or large T1 high-grade and/or recurrent high-grade T1 |  |  |  |  |
| 1. Persistent high-grade T1 disease on repeat resection |  |  |  |  |
| 1. Existence of lymphovascular invasion (LVI) |  |  |  |  |
| 1. Existence of variant histology |  |  |  |  |

1. For NMIBC patients with the stated characteristics, please select your preferred first, second and third choice treatment from the list below and indicate each of them accordingly in the table. An example is shown in the first row of the table.
2. Intravesical BCG for **1 year** with **three-weekly instillations** at months 3, 6, and 12 (after induction course).
3. Intravesical BCG for **3 years** with **three-weekly instillations** at months 3, 6, 12, 18, 24, 30, 36 (after induction course).
4. Intravesical BCG given **monthly** for up to **1 year** (after induction course)
5. Induction BCG without maintenance
6. Induction intravesical chemotherapy alone.
7. Intravesical chemotherapy given **monthly** for **6 months** (after induction course)
8. Intravesical chemotherapy given **monthly** for **1 year** (after induction course)
9. Radical cystectomy
10. Radiation therapy
11. Clinical trial, please specify
12. No treatment
13. Others, please specify

**For medical oncologist**

🖵 Opt-out from answering this question as I do not treat many of these patients in my practice

🖵 Opt-out from answering this question as not within my clinical expertise/ not qualified

| **Patient characteristics** | **(i) First choice** | **(ii) Second choice** | **(iii) Third choice** |
| --- | --- | --- | --- |
| *E.g. Patients with first occurrence of solitary low-grade Ta >3 cm* | *E* | *D* | *F* |
| 1. Patients with first occurrence of solitary low-grade Ta >3 cm tumor |  |  |  |
| 1. Patients with first occurrence of solitary low-grade Ta ≤3 cm tumor |  |  |  |
| 1. Patients with first occurrence of low-grade Ta, multifocal tumor |  |  |  |
| 1. Patients with first occurrence of high-grade Ta, ≤3 cm tumor |  |  |  |
| 1. Patients with first occurrence of high-grade Ta, >3 cm tumor |  |  |  |
| 1. Patients with first occurrence of low-grade T1 tumor |  |  |  |
| 1. Patients with first occurrence of high-grade T1 without CIS |  |  |  |
| 1. Patients with first occurrence of CIS alone |  |  |  |
| 1. Patients with first recurrence of single (BCG-naïve) low-grade Ta tumor (≤3 cm) |  |  |  |
| 1. Patients with first recurrence of single (BCG-naïve) low-grade Ta tumor (>3 cm) |  |  |  |
| 1. Patients with any high-grade T1 associated with concurrent bladder CIS |  |  |  |
| 1. Patients with any high-grade T1 with CIS in the prostatic urethra |  |  |  |
| 1. Patients with multiple and/or large T1 high-grade and/or recurrence of high-grade T1 |  |  |  |
| 1. Patients with persistent high-grade T1 disease on repeat resection |  |  |  |
| 1. Patients with lymphovascular invasion (LVI) |  |  |  |
| 1. Patients with variant histology |  |  |  |

1. For NMIBC patients with the stated characteristics and are assumed to be **fit for cystectomy**, please select your preferred first, second and third choice treatment from the list below and indicate the corresponding letter in the table. If the patient with the stated characteristics will be treated similarly to BCG-unresponsive patients, please indicate ‘Yes’ in the corresponding column (i). An example is shown in the table.
2. Radical cystectomy
3. No treatment
4. Systemic treatment in clinical trials, please specify
5. Radiation therapy

Intravesical treatment options

1. BCG re-treatment
2. Mitomycin
3. Gemcitabine
4. Doxorubicin
5. Epirubicin
6. Valrubicin
7. Docetaxel
8. Sequential gemcitabine/ docetaxel
9. Sequential gemcitabine/ mitomycin
10. Hyperthermic intravesical chemotherapy, please specify chemotherapy option
11. Electromotive drug administration, please specify chemotherapy option
12. Instiladrin
13. Bropirimine
14. MCNA: *Mycobacterium phlei* cell wall-nucleic acid complex
15. Anti-PD1/ PDL1
16. Others, please specify

**For medical oncologist**

🖵 Opt-out from answering this question as I do not treat many of these patients in my practice

🖵 Opt-out from answering this question as not within my clinical expertise/ not qualified

|  |  | **Preferred choice after having received adequate BCG treatment** | | |
| --- | --- | --- | --- | --- |
| **Patients who have received adequate BCG-treatment and have** | **(i) Are these patients considered as BCG-unresponsive patients? (Yes/No)** | **(ii) First choice** | **(iii) Second choice** | **(iv) Third choice** |
| E.g. *Single low-grade Ta (≤3 cm), multiple recurrences* | *No* | *F* | *G* | *H* |
| 1. Single low-grade Ta (≤3 cm), multiple recurrences |  |  |  |  |
| 1. Single low-grade Ta (>3 cm), multiple recurrences |  |  |  |  |
| 1. Multiple, first recurrent and large (>3 cm) low-grade tumor |  |  |  |  |
